# Optimizing metformin therapy in Saudi patients: The crucial role of vitamin B12 monitoring and medication adherence

**DOI:** 10.1016/j.jtumed.2026.02.003

**Published:** 2026-02-25

**Authors:** Mohamad B. Dahha, Gharam A. Alahmadi, Bassel M. Dahha

**Affiliations:** a Ibn Sina National College for Medical Studies, Jeddah, KSA; b Department of Internal Medicine, Nahdi Clinics, Jeddah, KSA

**Keywords:** Diabetic neuropathy, Medication adherence, Metformin, KSA, Type 2 diabetes, Vitamin B12 Deficiency

To the Editor,

We read with great interest the prospective cohort study published in the December 2025 issue of the *Journal of Taibah University Medical Sciences*, titled “**Efficacy of immediate-release versus extended-release metformin on glycemic control and insulin resistance in Saudi patients with type 2 diabetes**”.^1^ The authors are to be commended for providing valuable local evidence comparing these formulations, offering a practical guide for clinicians navigating the high prevalence of Type 2 Diabetes Mellitus (T2DM) in the Kingdom.

While the study's primary endpoint focused on glycemic parameters (HbA1c) and insulin resistance, we believe it is imperative to integrate a concurrent safety perspective regarding **Metformin-associated Vitamin B12 Deficiency (MAVD)**. The relationship between long-term metformin use and B12 depletion is well-established, yet frequently under-screened in primary care settings.

## The biochemical mechanism and local relevance

The mechanism of MAVD is thought to be calcium-dependent. Metformin creates a hydrophobic tail effect that interferes with the calcium-dependent binding of the Intrinsic Factor-Vitamin B12 complex to the cubilin receptor in the terminal ileum.^2^ This is particularly relevant in the Saudi context, where subclinical Vitamin B12 deficiency is already prevalent due to dietary habits and genetic predispositions. A landmark study conducted in **KSA** reported that approximately 30 % of diabetic patients on metformin had either deficient or borderline Vitamin B12 levels.^3^ Therefore, treating Saudi patients with high-dose metformin without periodic B12 monitoring may inadvertently precipitate a “double hit” of deficiency.

## Clinical implications: the neuropathy mimic

The clinical danger lies in the symptomatic overlap between Vitamin B12 deficiency and diabetic peripheral neuropathy. Both present with paresthesia and sensory loss. Without differentiating these etiologies, clinicians might intensify gabapentinoid therapy for “worsening diabetic neuropathy” when the root cause is actually a reversible vitamin deficiency induced by the very drug used to treat the diabetes.^4^ We strongly advocate that future local guidelines should mandate annual B12 screening for patients on high-dose metformin (>1500 mg/day) or those with a duration of use exceeding 4 years, aligning with the American Diabetes Association (ADA) recommendations.

## Adherence as a confounding variable

Furthermore, regarding the study's comparison of formulations, we propose that **“Medication Adherence”** plays a role as significant as pharmacokinetics. The extended-release (XR) formulation, typically requiring once-daily dosing, significantly reduces the pill burden compared to the multiple daily doses of the immediate-release (IR) form. Global data suggests that adherence rates can jump from ∼60 % to over 80 % when switching from IR to XR formulations.^5^ Thus, the superior glycemic control observed in the XR group may be largely attributable to the behavioral factor of compliance.

In conclusion, while we support the expanded use of extended-release metformin for its tolerability, we urge the medical community to adopt a holistic monitoring approach. Treating the “sugar” should not come at the cost of the “nerves.”

## Ethical approval

Not applicable (Commentary/Letter to Editor).

## Source of funding

No funding was received for this work.

## Conflict of interest

The authors declare no conflict of interest.
